# Applications of artificial intelligence in digital pathology for gastric cancer

**DOI:** 10.3389/fonc.2024.1437252

**Published:** 2024-10-28

**Authors:** Sheng Chen, Ping’an Ding, Honghai Guo, Lingjiao Meng, Qun Zhao, Cong Li

**Affiliations:** ^1^ School of Clinical Medicine, Hebei University, Affiliated Hospital of Hebei University, Baoding, China; ^2^ The Third Department of Surgery, the Fourth Hospital of Hebei Medical University, Shijiazhuang Hebei, China; ^3^ Hebei Key Laboratory of Precision Diagnosis and Comprehensive Treatment of Gastric Cancer, The Fourth Hospital of Hebei Medical University, Shijiazhuang, Hebei, China; ^4^ Big Data Analysis and Mining Application for Precise Diagnosis and Treatment of Gastric Cancer Hebei Provincial Engineering Research Center, Shijiazhuang, Hebei, China; ^5^ Department of Hepatobiliary Surgery, Affiliated Hospital of Hebei University, Baoding, China

**Keywords:** gastric cancer, machine learning, digital pathology, artificial intelligence, computational pathology

## Abstract

Gastric cancer is one of the most common cancers and is one of the leading causes of cancer-related deaths in worldwide. Early diagnosis and treatment are essential for a positive outcome. The integration of artificial intelligence in the pathology field is increasingly widespread, including histopathological images analysis. In recent years, the application of digital pathology technology emerged as a potential solution to enhance the understanding and management of gastric cancer. Through sophisticated image analysis algorithms, artificial intelligence technologies facilitate the accuracy and sensitivity of gastric cancer diagnosis and treatment and personalized therapeutic strategies. This review aims to evaluate the current landscape and future potential of artificial intelligence in transforming gastric cancer pathology, so as to provide ideas for future research.

## 1 Introduction

Gastric cancer (GC), a globally formidable health challenge, is the fifth most common cancer worldwide and the third leading cause of cancer-related mortality (1). This malignancy exhibits a marked geographical variation, with high incidence rates in East Asia, Eastern Europe, and parts of South America, while lower rates are observed in North America and Africa (2, 3). The high mortality rate associated with GC is primarily due to late-stage diagnosis and the complexity of the disease, which present significant hurdles in both diagnosis and treatment (4, 5). The global impact of gastric cancer is profound, affecting millions of individuals and their families (6). GC frequently progresses silently, with many patients remaining asymptomatic until reaching advanced stages (7). Symptoms, such as weight loss, abdominal discomfort or nausea, are vague and nonspecific, and may delay medical assistance and diagnosis (8, 9). Early detection is crucial because it significantly improves patient survival (10). However, the absence of distinctive symptoms and the lack of robust screening procedures lead to delayed diagnosis (11). The management of GC generally comprises a multidisciplinary approach, including surgery, chemotherapy, radiation therapy, and immunotherapy. However, the efficacy of these treatments is contingent upon the stage of the disease at the time of diagnosis (12, 13). The heterogeneity of GC, with its various histological and molecular subtypes (14), introduces additional complexity to its management, underscoring the necessity for precise diagnostic and therapeutic strategies (15).

Histopathologic examination has long been the cornerstone in cancer diagnosis (16, 17). Traditional pathology involving microscopic examination of tissue samples has limitations in terms of scalability, speed and objectivity (18, 19). Digital pathology involves converting glass slides into superresolution digital images that can be easily stored, shared and analyzed (20, 21). This transformation has potentially altered the operational paradigms of pathologists, facilitating more effective collaboration and laying the groundwork for the incorporation of sophisticated computational technologies (22).

Artificial intelligence (AI) has revolutionized various fields, including pathology, by enabling advanced data analysis (23, 24). The potential capabilities of AI are particularly useful for identifying subtle or complex features and specific pathological conditions that might be challenging for a human pathologist to discern (25–28). In gastric cancer, AI could analyze digital histopathological images to aid in diagnosis, prognostication, and treatment decisions, offering critical insights, precise tumor classification, grading, and potentially predicting therapy responses (29).

## 2 Fundamentals of AI in digital pathology

Digital and computational pathology convert glass slides into digital images using high-resolution scanners, enabling viewing, management, and analysis on computers (30). This transition offers several advantages, such as easy sharing with experts globally, facilitating remote consultations, promoting standardized interpretations, and reducing inter-observer variability (31–33). Early pathology image analysis efforts, like those by Prewitt and Mendelson in 1966, were limited by scarce computational resources and simple algorithms (34). With the rise of AI and machine learning (ML), more powerful computing enabled sophisticated analyses, such as classifying types of cancer or predicting disease outcomes (35). Another breakthrough came in the 2010s with deep learning (DL), particularly CNNs, which automatically learn features from raw data, eliminating the need for manual selection (36). The potential of DL in computational pathology was driven by the availability of large annotated datasets (37).Initiatives like The Cancer Genome Atlas (TCGA) and public competitions provided essential data for training and validating models, accelerating progress in the field (38, 39).

**Figure 1:**
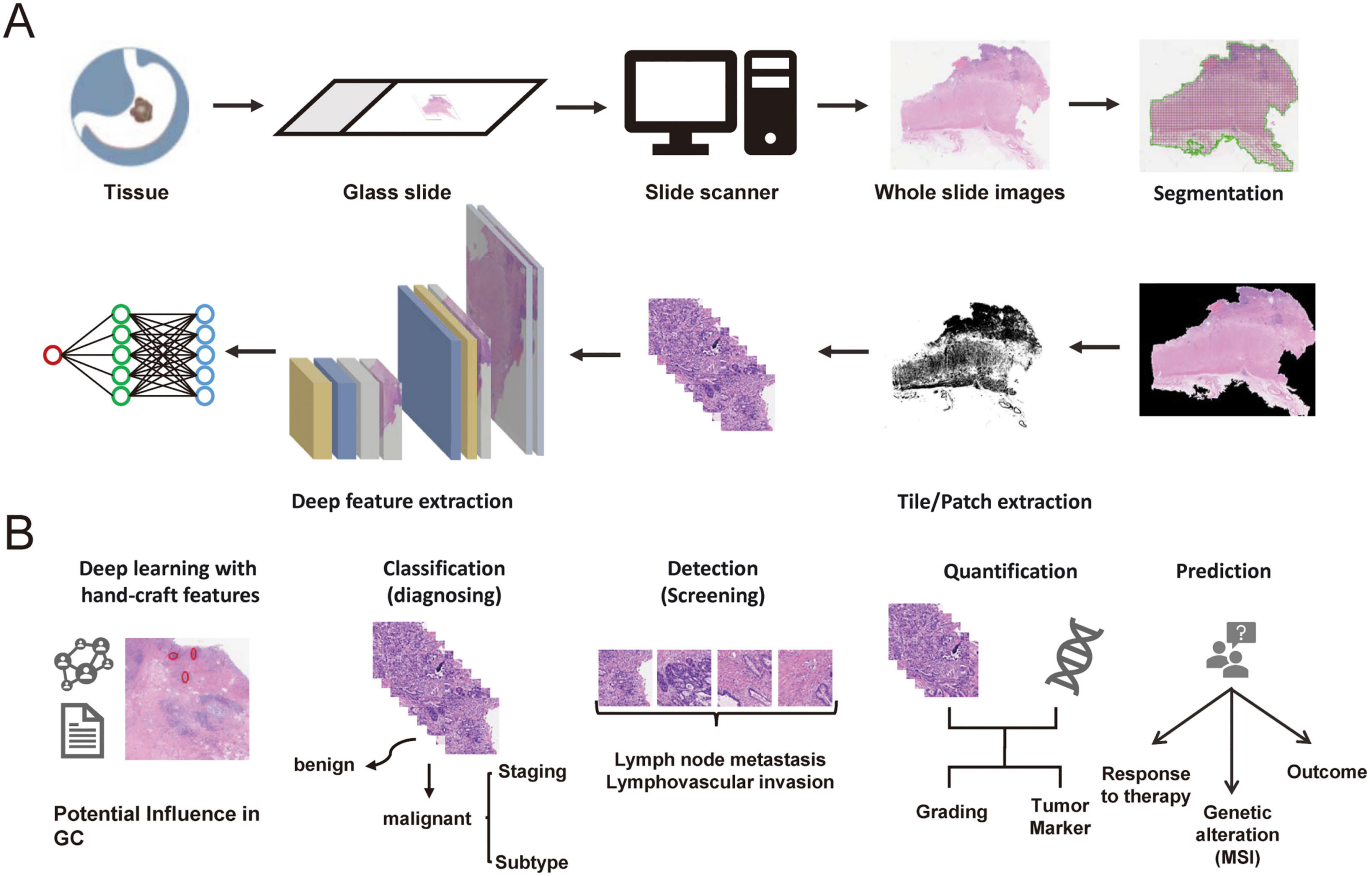
**(A)** The digital pathology workflows of AI for gastric cancer. **(B)** The potential clinical applications of AI in digital pathology for gastric cancer.

The applications of AI in GC pathology are vast and transformative, promising for enhancing various aspects of pathology practice from diagnostics to research, including automated disease diagnosis, tumor detection and quantification, grading and staging cancer, predictive analytics for prognosis, treatment response, biomarker identification and data integration for holistic analysis (**Figure 1**).

## 3 Application of AI in the detection and pathological diagnosis of GC

GC is the prevailing malignancy in humans, emphasizing the paramount importance of early diagnosis to enhance cure rates and prognoses (40). Currently, pathological diagnosis continues to serve as the definitive method for diagnosing gastric cancer (41). Within the diagnostic procedure, the discrimination between benign and malignant lesions constitutes a pivotal and indispensable step. The application of AI in the detection and pathological diagnosis of GC are summarized in **Table 1**. Yoshida et al. proposed an image analysis software called “e-Pathologist,” which was the first application of an AI algorithm to categorize gastric biopsy Whole slide images (WSIs) as carcinoma, adenoma, or non-malignant. The sensitivity, detection specificity, negative predictive value, and positive predictive value of the e-Pathologist test were 90%, 50%, 99.8%, and 1.7% respectively (42). Abe et al. (43)designed an artificial intelligence‐based system grounded in a deep convolutional neural network (DCNN) for diagnosing gastric biopsies (normal, carcinoma) through the use of H&E-stained WSIs. Their AI-system was tested on validation cohort including 3450 gastric biopsy samples of 1772 patients from 10 different institutes, with an accuracy of 91.3%. This shows the potential power of AI-based model in alleviating the pressure on pathologists in diagnosing gastric biopsies. In a similar study, Fan and colleagues (44) applied a DL model for automated gastric endoscopic biopsy classification. Five common CNN models (VGG-16, VGG-19, ResNet-50, Xception, and InceptionV3) were utilized for the Japanese “Group Classification” of Gastric Carcinoma. The ResNet-50 model demonstrated the best performance, with an accuracy of 93.16%. Likewise, Iizuka et al. (45) trained a CNN model based on the inception-v3 framework for stomach biopsy whole slide image (WSI) classification into three types: adenocarcinoma, adenoma, and non-neoplastic. Then a test cohort of 45 WSIs was used to confirm this model, and an accuracy of 95.6% and an AUC of 0.924 were obtained. These indicate the efficacy of AI model in classifying epithelial lesions in biopsy WSIs of gastric.

**Table 1:** Characteristics of the application of AI for the detection and pathological diagnosis in gastric cancer.

Author	Year	Country	AI Model	Specimen type	Training and Validation Data Set/WSIs/No. of Patients (n)	Aim of Classification	Pixel Levels	Performance Metrics	External Validation Dataset/WSIs/No. of Patients (n)	External Validation Result	Ref.
Yoshida	2018	Japan	NC	biopsies	In-house/3062/3062	carcinoma, adenoma, or no malignancy	1024×1024	overall concordance rate: 0.556	NS	NS	(42)
Abe	2022	Japan	GoogLeNet	biopsies	In-house /4511/984	Japanese Classification	224×224	accuracy: 0.910	In-house/3450/1772	accuracy: 0.946	(43)
Fan	2022	China	ResNet50	biopsies	In-house /260/173	Japanese Classification	mixed size∗	accuracy: 0.932 AUC: 0.994	NS	NS	(44)
Iizuka	2020	Japan	inception-v3	biopsies	In-house/4128/NC	ADC, adenoma, and non-neoplastic	512×512	AUC: 0.980	In-house /500/NC, TCGA-STAD/475/NC	AUC: 0.974 AUC:0.924	(45)
Ko	2022	Korea	DenseNet201	biopsies	In-house/1762/NC	NFD, Dysplasia, and Malignant	256×256	accuracy:0.960	NS	NS	(46)
Park	2021	South Korea	NC	surgical sections biopsies	In-house/2434/2278	adenomas and carcinomas	NC	AUC: 0.995 Specificity: 0.981 Sensitivity: 0.991	In-house/7432/5379	AUC: 0.979, Specificity: 0.975, Sensitivity: 0.967	(47)
Sharma	2017	Germany	self-designed CNN	surgical sections	In-house/NC/454	HER2+ tumor, HER2− tumor and non-tumor	512×512	accuracy: 0.699	NS	NS	(48)
Sharma	2017	Germany	self-designed CNN	surgical sections	In-house/NC/454	Necrotic and non-necrotic.	512×512	accuracy: 0.814	NS	NS	(48)
Wang	2019	China	RMDL	NC	In-house/608/NC	normal, dysplasia, and cancer	NC	accuracy: 0.865	NS	NS	(49)
Song	2020	China	DeepLab v3	surgical sections biopsies	In-house/2123/1500	malignant and benign	320×320	AUC: 0.945 accuracy: 0.833	In-house /595/355 In-house /987-541	AUC:0.990 AUC:0.996	(53)
Lan	2023	China	DeepLabv3+	surgical sections biopsies	In-house/1668/1294	malignant and benign	512×512	accuracy: 0.918	NS	NS	(55)
Tung	2022	China	YOLOv4	biopsies	In-house/NC/50	malignant and benign	1024×1024	sensitivity: 0.966 specificity: 0.896	NS	NS	(60)
Zhu	2022	China	DCNN, GCN	biopsies	In-house/2618/NC	WHO classification	512 × 512	NS	In-house /2003/NC	AUC: 0·979	(61)
Yong	2023	Malaysia	EfficientNetB0	NC	GasHisSDB/600/NC	normal and abnormal	80×80	accuracy: 0.968	NS	NS	(62)
			DenseNet169				120×120	accuracy: 0.982			
			DenseNet121				160×160	accuracy: 0.991			
Veldhuizen	2023	Germany	ResNet-50	NC	TCGA-GC/166/166	Lauren classification	224 x 224	AUC:0.930	In-house/361/361 In-house/251/251	NS	(65)
Kanavati	2021	Japan	InceptionV3	biopsies	In-house/2919/NC	ADC, adenoma, and non-neoplastic	512×512	average AUC: 0.97	NS	NS	(66)
Tsuneki	2022	Japan	EfficientNetB1	ESD	In-house/1150/NC	Differentiation grade	224×224	NS	In-house/719/NC	AUC:0.975	(67)
Fu	2022	China	StoHisNet	NC	SEED/4290/NC	normal, tubular ADC, mucinous ADC, and papillary ADC.	224×224	accuracy:0.947	NS	NS	(68)
Ning	2023	China	NC	surgical sections	In-house/70/70	Differentiation grade	1024×1024	AUC:0.910	NS	NS	(69)
Su	2022	China	ResNet-18	surgical sections	In-house/467/467	Differentiation grade	224×224	accuracy:0.986	NS	NS	(70)

NS, not specified. NC, not clear.

In a different study, Ko et al (46) proposed a rapid daily post-analytical AI-assisted quality control (QC) system for pathologists to evaluate stomach biopsy specimens. The QC system was composed of laboratory information and an AI WSI-classifier model based on the DenseNet algorithm. The AI WSI-classifier distinguished histopathological WSIs of biopsy specimens among three classes: negative for dysplasia, dysplasia, and malignant, and demonstrated high accuracy rates (95.8%) from their internal verification set, which included 150 gastric biopsy WSIs. Evidence like this demonstrates the clinical efficacy of AI algorithms in enhancing the diagnostic accuracy and consistency of GC. Park et al. (47) constructed a DL formula to classify gastric biopsy lesions into dysplasia, tubular adenoma, and carcinoma. The formula model obtained an accuracy of 97% with 100% sensitivity and 97% specificity in distinguishing gastric epithelial tumors. This showcases the aid of AI system could decrease the missed diagnosis of cancer, especially in biopsies with small areas of lesion. A dataset comprising 640 H&E-stained histopathological slides of GC tissues and immunohistochemistry (IHC) for the HER-2 gene was utilized by Sharma et al (48). They developed a CNN system capable of swiftly and accurately distinguishing between tumor and non-tumor regions in HE-stained WSIs. Compared to traditional methods and the AlexNet architecture, this model exhibited superior discrimination of necrotic tumor areas, achieving an overall accuracy of 0.8144. This highlights the potential of AI system in cancer classification and necrosis detection without immunohistochemical staining. Wang et al. (49) developed a two-stage deep learning model to automatically classify WSIs of gastric tissue lesions into normal, dysplasia, and cancer categories, and validated the accuracy at 86.5% using 200 WSIs in the testing set. The core strength of RMDL lies in its instance recalibration module, which autonomously identifies crucial instances for image-level forecasting. However, the application of AI model with a two-stage algorithm may significantly influence on pathologists in diagnosing cancer, which neglected the potential interaction between local tiles.

With the creation of exceptionally efficient algorithms, some pathology laboratories are adopting the routine utilization of digital slides in the format of WSIs as part of their daily diagnostic procedures (50–52). Song et al. (53) proposed a CNN of the DeepLab v3 network trained with 2123 pixel-level gastric cancer WSIs from 1500 GC patients, and the performance of the model exhibited nearly 100% sensitivity and 80.6% specificity for gastric carcinoma detection on 3,212 WSIs in the test cohort. It may assist pathologists in enhancing diagnostic precision and averting misdiagnoses in their everyday tasks. In another study, they assessed the support provided by the DL model in assisting pathologists with diagnosing gastric carcinoma, through the design of a comprehensive multireader multicase examination (54). The results showed that the assistance of DL model indeed enhanced the accuracy and efficiency of pathologists in distinguishing between malignant and benign lesions. However, with the assistantance of the DL model, the specificity of GC detection remains unaffected. In another analogous investigation, Lan et al. established a DL-driven pathological auxiliary diagnostic system based on 2,020 stomach H&E-stained WSIs (55). With the assistance of this system, pathologists experience a substantial decrease in the average false-negative rate and average false-positive rate and a reduction in the time of diagnosis. The incorporation of AI in disease diagnosis necessitates a careful and considered approach (56, 57). The consequences of false positive and false negative cases have far-reaching implications for patient welfare and should not be underestimated (58, 59).

In the creation of simple AI-assisted diagnostic system, attaining a minimal false-negative rate holds the same significance as ensuring a high level of accuracy. Tung et al. (60) constructed a DL algorithm based on the YOLOv4 network structure to identify GC regions from endoscopic biopsy WSIs, and obtained a detection accuracy of 91%, with a sensitivity of 96.6% and a specificity of 89.6%. The advantageous features of this system are its ability to inspect all GC regions in an image and reduced false-negative rate. Zhu et al. created an endoscopic gastric biopsy assistant system bonding DCNN and graph convolutional networks for assisting pathologists in delineating crucial regions for diagnosis in gastric biopsy WSIs (61). This highlights the possibility and advantages of using AI system in the clinical routine work in routine practice scenarios. In 2023, Yong and colleagues propounded an assemblage DL model built uponbtransfer learning principles from multiple pre-trained architectures, including MobileNet, DenseNet, EfficientNet, InceptionV3, and Xception for gastric lesion categorization (62). The observations revealed that the ensemble model attained an advanced accuracy ranging from 97.72 to 99.20% which was verified on lower resolution WSIs from GasHisSDB. This demonstrates the potential of AI in classifying the pathological low-resolution images for cost reduction.

In intricate pathological assessments, gastric lesions should be subdivided into different histological subtypes rather than simply categorized as malignant or benign (63). As a highly heterogeneous disease, GC could be divided into two primary histological subcategories within the Lauren classification system (64). Veldhuizen et al. (65) sought to develop a classifier based on attention-based deep multiple-instance learning to distinguish between intestinal and diffuse type GC WSIs. The results demonstrated that the classifier achieved strong discriminatory performance (mean AUROC 0.93 ± 0.07). Furthermore, in comparison to the pathologist-based Lauren classification, the DL-based classifier exhibited improved stratification of the 5-year survival rates for GC patients. However, in two independent external validations, the DL-classifier performed unsatisfactorily. There are 54% of the samples that the DL-classifier identified as intestinal type while pathologist initially classified as diffuse type. These results showed that the progression of AI may enhance the diagnostic accuracy among pathologists and reduce observer disagreement in GC histological classification. In a similar study, GC histological patterns were categorized into diffuse-type adenocarcinoma (ADC) and others on H&E-stained slides using a CNN framework to train four individual models. The best model achieved the AUROC of 0.95–0.99 in five validation groups (66). Another gastric ADC classification DL model was developed by Tsuneki et al. and achieved an AUROC of 0.975 for classifying endoscopic submucosal dissection (ESD) WSIs into poorly differentiated ADCs and others (differentiated and non-tumor) (67). These show the promise of AI in accurately distinguishing poorly differentiated adenocarcinoma for improved clinical decision-making and outcomes. In a different study, Fu et al. created a novel architecture, StoHisNet, combining transformer and a CNN to classify GC WSIs into four subtypes (tubular, mucinous, and papillary adenocarcinoma, non-tumor), with a classification accuracy of 94.69% (68). Ning et al. utilized the U-Net algorithm and QuPath software to identify differentiated and undifferentiated lesions in WSIs of mixed-type GC (69). Finally, in the research of Su et al., a CNN-based DL-model was established to recognize the degree of tumor differentiation and status of microsatellite instability (MSI) in GC HE-stained WSIs, achieving F1 values of 0.86 and 0.89 for poor-differentiated ADC and well-differentiated ADC sorting, respectively and an accuracy of 83.87% for predicting MSI status (70).

## 4 Application of AI in molecular phenotype prediction of GC

Histological classification has been shown to be insufficient for identifying actionable molecular targets (71). In acknowledgment of limitations, extensive molecular profiling has given rise to a variety of classifications based on molecular characteristics to uncover the clinical features and biological characteristics to inform therapeutic decision-making and prognosis of GC patients (72–75). Harnessing AI holds the potential to ease the burden of molecular property testing to alleviate the pressure on pathologists (76–79). The application of AI in molecular phenotype prediction of GC are summarized in **Table 2**.

**Table 2:** Characteristics of the application of AI in molecular phenotype prediction of gastric cancer.

Author	Year	Country	AI Model	Specimen type	Training and Validation Data Set/WSIs/No. of Patients (n)	Aim of study	Pixel Levels	Performance Metrics	External Validation Dataset/WSIs/No. of Patients (n)	External Validation Result	Ref.
Kather	2019	Germany	ResNet18	NC	TCGA/315/315	predicting MSI status	224x224	AUC:0.81	In-house/NC/185	AUC:0.89	(81)
Muti	2021	Germany	shufflenet	surgical sections	9 In-house,TCGA/NC/2823	predicting MSI status	512x512	AUC:0.745	NS	NS	(82)
					9 In-house,TCGA/NC/2823	predicting EBV status		AUC:0.810			
Zhu	2022	China	ResNet-18	NC	TCGA/285/285	predicting MSI status	224x224	AUC:0.80	NS	NS	(83)
Lee	2022	South Korea.	Inception-v3	NC	TCGA/NC/331	predicting MSI status	360×360	AUC:0.902	In-house/NC/383	AUC:0.874	(84)
Wang	2022	Australia	EfficientNet-b1	NC	TCGA/332/295	predicting MSI status	224x224	AUC:0.898	NS	NS	(85)
						predicting EBV status		AUC:0.764			
Schmauch	2020	USA	ResNet50	NC	TCGA/323/NC	predicting MSI status	224x224	AUC: 0.76	NS	NS	(86)
Jeong	2022	Korea	InceptionV3	NC	TCGA, in-house/427/NC	predicting EBV status	512×512	ACC:0.99	In-house/60/NC	ACC:0.92	(88)
Zhang	2021	USA	Resnet18	NC	TCGA/NC/122	predicting EBV status	512×512	AUC:0.85	NS	NS	(89)
Vuong	2022	Korea	EfficientNet	surgical sections	In-house/24/NC	predicting EBV status	256×256	ACC:0.937	In-house/286/286	ACC:0.947	(90)
Zheng	2022	China	EBVNet	surgical sections	In-house/1006/727	predicting EBV status	512x512	AUC:0.969	In-house/417/417	AUC:0.941	(91)
									TCGA/258/239	AUC:0.895	
Jang	2021	South Korea	Inception-v3	NC	TCGA/NC/NC	predicting gene mutation	360x360	AUC:0.661-0.858	In-house/NC/96	AUC:0.597-0.666	(92)
Saldanha	2022	Germany	RetCCL	surgical sections	In-house/NC/1210	predicting MSI status	NC	NC	TCGA/NC/351	AUC: 0.809	(93)
					In-house/NC/1211	predicting EBV status	NC	NC	TCGA/NC/351	AUC:0.837	

NS, not specified. NC, not clear.

With the advancement of AI technology, it has demonstrated promising results and the potential to predict MSI status in a cost-effective manner (80). In 2019, Kather et al. yielded the first entirely automated DL system based on the Resnet-18 framework to infer MSI status from GC histopathological images, with an AUC of 0.81 in the TCGA cohort and an AUC of 0.69 in the external validation cohort (81). Molecular phenotype prediction using AI and pathomics holds significant promise for enhancing the early management of GC. Subsequently several similar studies integrating advanced technical DL algorithms have provided further insights into the efficacy of ML for predicting MSI status in GC histological WSIs, and the model exhibited performances with AUCs ranging from 0.54 to 0.90 (82–86). This demonstrates the effectiveness of AI algorithms in accurately differentiating MSI status. AI has been applied widely to predict molecular categories directly from GC H&E-stained slices and exhibited equivalent performance compared with identification staining methods noticed on WHO rules (87). Such evidence highlights the potential of AI in areas like molecular identification and phenotype prediction, paving the way for advancements in personalized medicine.

EBV-associated GCs are recognized as molecularly and pathologically distinct from those EBV-negative. Numerous studies have demonstrated the efficacy of AI in predicting Epstein–Barr virus (EBV) status. Jeong et al., trained a DL classifier to detect the EBV status from H&E stained digital WSIs based on the InceptionV3 architecture, with the accuracy of 0.92 (88). In another research, a ResNet18-based CNN framework was adopted to identify EBV status through GC H&E-stained WSIs and obtained an AUC of 0.85 (89). In line with these findings, Vuong et al., first proposed a CNN model based on EfficientNet to evaluate the EBV status of H&E–stained WSIs from biopsy specimens of GC, and achieved admirable performance with an accuracy of 0.938 (90). This showcases the potential of AI in analyzing intricate patterns in medical imaging and discovering virus infection status. Moreover, Zheng et al. (91), proposed a human−machine collaboration approach that combined a DCNN model and pathologists for EBV prediction. With the collaboration of pathologists, the model attained the AUC ranging from 0.945 to 0.969 for predicting GC EBV status. This suggests the potential of human-AI fusion strategy in identifying EBV status and its role in revealing new insights into identify complex molecular subtypes.

AI systems offer a cost-effective and time-efficient alternative for detecting gene mutations from histologic image (92). A multistep CNN networks based on the Inception-v3 architecture were trained to assess the mutational status of 5 genes (CDH1, ERBB2, KRAS, PIK3CA, and TP53) in GC, with CDH1 and PIK3CA exhibiting the highest accuracies of 0.847 and 0.834, respectively, for frozen WSIs and KRAS and CDH1 having the best accuracies of 0.894 and 0.820, respectively, for formalin-fixed paraffin-embedded tissue WSIs (93). This indicates the potential of AI in identifying genetic alterations from H&E-stained slides, potentially offering an alternative from genomics research.

However, due to high tumor heterogeneity, DL-based models seem to have poorer performance in GC than in other carcinomas (94). To address these challenges, swarm learning (SL), a decentralized ML robust, was employed to acquire highly predictable AI-classifiers for MSI and Epstein–Barr virus (EBV) status prediction in GC digital WSIs (95). These studies collectively demonstrate analyzing the morphology of a lesion and its microenvironment by AI could improve the accuracy of molecular phenotype predictions, potentially resulting in more personalized therapeutic approaches, particularly beneficial in resource-limited settings. Although the potential to implement molecular phenotype prediction from digitized H&E-stained tissue is promising, substantial validation is still necessary to determine the clinical utility of AI-pipelines.

## 5 Application of AI in determining GC prognosis

### 5.1 Prediction of lymph node metastasis

Lymph node metastases (LNM) stands as a key prognostic indicator for individuals diagnosed with GC (96, 97). Pathologically evaluating lymph nodes plays an essential role in ascertaining clinical staging and directing treatment interventions (98, 99). AI-based algorithm has the potential to alleviate pathologists’ workload while enhancing diagnostic accuracy. Currently, only a few studies have explored the application of artificial intelligence in evaluating LNM in lymph node WSIs. DL methods could extract information not only to predict LNM and patient outcomes from primary GC tissue (100) but also to explore prognostic features from WSI from LNs (101). Hu et al. proposed a cascade DL-based system for detecting and quantifying LNM in WSI of LNs, which achieved effectively performance for patients after neoadjuvant therapy (102). This underscores the effectiveness of the AI approach for automated lymph node metastatic quantification and identification. In the similar study, Huang et al. introduced an enhanced streaming CNN algorithm trained on gigapixel images of LN-level WSIs to detect LNM. The results showed that with the assistance of the AI model, the sensitivity of detecting micrometastases, and isolated tumor cells have been increased, with a less review time. However, in another study, different results were observed. The AI-diagnostic model has the potential to enhance the sensitivity of pathologists in identifying micrometastases, but the review time has extended (103). In comparison to manually review LNs microscopically, AI-based digital pathology systems provide easier input acquisition, greater diagnostic accuracy, and better scalability for clinical application.

Lymphovascular invasion (LVI) is a histomorphological feature indicating LNM in primary GC tissues (104, 105). In 2023, Lee et al. proposed an ensemble DL algorithm based on the ConViT and YOLOX architectures for detecting LVI foci from GC histopathology images, achieved an AUC of 0.9438 in the external validation cohort (106). In a similar study, hard negative mining algorithm was used to develop a DL model for lymphatic invasion screening with GC digital WSIs (107). In summary, the design of AI in identifying metastatic cells opens avenues for reviewing LNM and managing gastric cancer patients.

### 5.2 Prediction of survival and therapeutic responsiveness

The function of AI should not be confined to replicating pathologists’ assessments. Some studies indicated that the significance of AI has the capability to predict patient outcomes from histopathological slides. Huang et al. trained a CNN model, MIL-GC, for predicting the outcomes of patients with GC by analyzing WSIs (108). This stratification is crucial as it correlates with different survival rates, thereby providing prognostic insights that can inform clinical decision-making. Some studies have explored the connection between cancer pathological features and treatment responsiveness (109, 110). Chen et al. proposed a signature to predict GC patient outcomes and adjuvant chemotherapy responses based on pathomic features extracted from H&E-stained images via AI-based image analysis techniques (111). Zhou et al. applied three DL algorithms to create an ensemble model for predicting the effectiveness of neoadjuvant chemotherapy from WSIs of GC patients (112). In another investigation, a DL-based network and corresponding digital pathology signature score were presented from WSIs for the assessment of GC patient outcomes and adjuvant treatment (113). These advanced AI models aid in the nuanced understanding of GC pathology, leading to better-informed prognosis and management plans.

### 5.3 Prediction of cancer recurrence

Despite the implementation of multimodal treatment strategies, the recurrence of GC remains a prevalent issue (114). Consequently, numerous ongoing studies are dedicated to identifying individuals at risk of recurrence following treatment. In 2023, the first pathomic signature was developed to predict peritoneal recurrence in GC patients with serosal invasion from digital H&E-stained images (115) by handcrafted feature-based approach. However, this approach could be both complex and time-consuming to use. Zhang et al. introduced a novel graph neural network, AGCNet, designed for predicting cancer recurrence by analyzing cancerous tissue WSIs. The network demonstrated notable effectiveness with an accuracy of 81.81% in bladder cancer, 69.66% in pancreatic cancer, and 81.96% in GC (116). AI as a collaborative tool offers a promising approach for cancer recurrence prediction and expands the application of digital pathology images.

## 6 Application of AI in GC immuno-oncology

Immuno-oncology is a field focused on the interaction between tumors and the immune system, delving into harnessing the immune system’s ability to combat cancer (117). Over the past three decades, remarkable progress has been made, underpinned by the emergence of innovative immunotherapies and their clinical triumphs, which have significantly revolutionized cancer treatment paradigms, such as immune checkpoint inhibitors, and CAR-T cell therapy (118). Researchers have extensively explored the application of AI in forecasting the effectiveness of immunotherapy (119). In 2023, Wei et al., constructed a DL-based stratification system by analyzing GC WSIs to identify molecular phenotypic features associated with immunotherapy responses, including molecular subtypes, immune checkpoints, genetic mutations, and the intricacies of signaling pathways (120). AI-based pathomic methods emerge as predictors of immunotherapy response, offering valuable insights for making informed treatment decisions. The tumor mutational burden (TMB) is a pivotal predictive biomarker, associated with the effectiveness of immunotherapy in GC patients (121). Li et al., developed a DL multimodal fusion model that combined GC WSI features with omics information for TMB prediction, with a notable AUC of 0.971 (122). This study demonstrated that multimodal approaches could enhance the efficiency of prediction models compared to unimodal algorithms, representing a promising direction for future AI-pathology research.

## 7 Application of large language models and generative AI in pathology

With the rise of large language models (LLMs) and the broader field of generative AI, computational pathology is poised to enter a new frontier. LLMs and generative AI can assist in extracting crucial information and automatic generating pathology reports (123). Choi et al., demonstrated that following LLMs to extract structured information from pathology reports can save significant time and costs compared to manual methods (124). In another study, ChatGPT was used to detect adenomas in 100 colorectal polyp photomicrographs, with sensitivity of 74% and specificity of 36% (125). This indicates the promise of LLMs in areas like diagnosis, though its limitations, emphasizing the need for expertise in pathology. “PathChat”, a Generative AI Copilot, was presented for assisting human pathology (126).It has the potential to significantly impact pathology education, research, and human-in-the-loop clinical decision-making. While the initial results are promising, there are abundant opportunities for further growth. The path forward in exploring LLMs and generative AI applications within the field of pathology is both challenging and intriguing.

## 8 Future directions and perspectives

The use of AI in gastric cancer (GC) pathology is showing a trend of dynamic growth worldwide (127, 128). In this review, we have outlined the primary research directions in the application of AI to GC pathology. The incorporation of AI has commenced demonstrating promising outcomes in augmenting diagnostic precision, forecasting patient prognoses, and uncovering innovative therapeutic targets in GC. The published data generally suggest that the application of AI in oncological histopathology could become feasible for routine clinical workflows in the foreseeable future (129, 130). However, several challenges need to be addressed to fully realize the potential of AI in this field (131).

Firstly, the demographic differences in GC prevalence must be considered. AI models developed and trained on datasets from specific populations may not perform well on others. Therefore, it is crucial to ensure diverse and representative training datasets that encompass various demographic and ethnic backgrounds. Histopathological heterogeneity presents another layer of complexity. GC can exhibit significant variability in their histological and molecular characteristics, which complicates the development of generalizable AI models. To tackle this, integrating multi-modal data analyses, combining histopathological information with other fields such as radiomics (132, 133), genomics (134) and proteomics, could provide a more holistic view for improving model accuracy and patient-specific predictions.

Additionally, one of the primary issues is the lack of transparency in AI models, often referred to as the “black box” problem, making it difficult to interpret their decisions directly (135, 136). Developing explainable AI (XAI) methods such as ablation study, visualization, that provide insights into how AI models arrive at their conclusions is essential. These methods can help bridge the gap between AI and clinical practice, ensuring that AI tools are both reliable and interpretable. Another challenge is data scarcity. The development of robust AI models requires large datasets, which are often unavailable, especially for rare cancer subtypes. Federated learning, a decentralized approach where models are trained across multiple centers without sharing patient data, could support collaborative efforts across institutions to share and pool data and overcome data scarcity while preserving privacy. For AI tools to be widely adopted, Ethical and legal Considerations must be seamlessly integrated into clinical workflows. This involves developing intuitive interfaces and ensuring that AI systems can work alongside existing diagnostic tools. Conducting clinical trials and pilot studies will be essential to validate the effectiveness of AI in real-world settings.

And to ensure protection of patient data, robust encryption, strict access control, data anonymization, and continuous monitoring must be implemented, in compliance with regulations like HIPAA and GDPR. Anyone aiming to develop or implement AI solutions in clinical practice must comply with the strict regulatory controls and safeguards (137, 138). And AI-model performance validation should be a dynamic and continuous effort, focused on maintaining the quality and reliability of the results provided by AI tools. Therefore, regular audits are recommended to identify and correct any biases within AI systems (139). Furthermore, the advancement of AI in pathology in clinical practice also require ongoing collaboration between pathologists, data scientists, software engineers, and regulatory bodies. Such interdisciplinary efforts are crucial for overcoming technical challenges and ensuring that AI tools are developed with clinical applicability in mind. Finally, The potential of AI in the field of GC histopathology has not yet been fully realized, such as dissecting mutation prediction (140), and developing pan-cancer AI-foundation models for cancer detection and biomarker prediction (141), remain under explored.

## 9 Conclusion

In conclusion, this review summarizes the applications of AI in the diagnosis, histopathological classification, prognosis, and treatment response assessment of GC. AI holds significant potential to address the challenges of objectivity and inter-observer variability in histopathology, underscoring the importance of further research to facilitate its integration into routine clinical practice for GC patients.
